# Investigating risk factors behind piglet facial and sow teat lesions through a literature review and a survey on teeth reduction

**DOI:** 10.3389/fvets.2022.909401

**Published:** 2022-12-02

**Authors:** Jen-Yun Chou, Jeremy N. Marchant, Elena Nalon, Thuy T. T. Huynh, Heleen A. van de Weerd, Laura A. Boyle, Sarah H. Ison

**Affiliations:** ^1^Pig Development Department, Animal and Grassland Research and Innovation Centre, Teagasc, Fermoy, Ireland; ^2^Institute of Animal Welfare Science, University of Veterinary Medicine, Vienna, Austria; ^3^Department of Clinical Studies, Swine Teaching and Research Center, School of Veterinary Medicine, University of Pennsylvania, Philadelphia, PA, United States; ^4^United States Department of Agriculture-Agricultural Research Service, Livestock Behavior Research Unit, West Lafayette, IN, United States; ^5^Eurogroup for Animals, Brussels, Belgium; ^6^Farm Technology, Department of Biosystems Engineering, Wageningen University and Research, Wageningen, Netherlands; ^7^Cerebrus Associates Ltd., Godalming, United Kingdom; ^8^World Animal Protection International, London, United Kingdom

**Keywords:** pig welfare, painful procedure, pig health, udder wound, skin laceration, teat fight

## Abstract

**Introduction:**

Piglet facial and sow teat lesions are the main reported reasons why pig producers routinely practice teeth resection. This is a painful procedure performed on piglets, where their needle teeth are clipped or ground to resect the pointed tip. The practice raises welfare concerns. In contrast to other procedures, such as tail docking, we know little about the risk factors for these two types of lesions.

**Methods:**

We employed two methods to answer these questions: (1) reviewing the literature to identify potential risk factors, and (2) surveying pig production stakeholders worldwide to identify the occurrence of these lesions and the strategies used in practice that enable pig producers to manage or prevent these lesions while avoiding teeth resection. For the literature review, we used Google Scholar to include peer-reviewed publications and gray literature. We distributed the survey using convenience sampling and documented information on the current situation regarding teeth resection, including the methods, frequencies, and reasons for resecting piglets' teeth, the occurrence of piglet facial and sow teat lesions, and measures used to prevent and control these lesions.

**Results:**

The literature review identified six major risk factors for both lesions, including the presence or absence of teeth resection, housing system, litter size, piglet management, environmental enrichment, milk production and other piglet management practices. However, most studies focused on the effects of the first two factors with very few studies investigating the other risk factors. There were 75 responses to the survey from 17 countries. The survey showed that half of the respondents practiced teeth resection with many recognizing that facial and teat lesions are the main reasons behind this practice. However, many producers used other interventions rather than teeth resection to prevent these lesions. These interventions focused on improving milk production of the sow, managing large litters, and providing environmental enrichment.

**Discussion:**

More research is needed to validate these interventions and more science-based advice is needed to bridge the gap between research and practice to help more producers further understand the cause of piglet facial and sow teat lesions to transition toward the cessation of routine teeth resection.

## Introduction

Piglets are born with eight fully erupted needle teeth (also known as “corner teeth”, including the canine and third incisor teeth in the left/right, upper/lower quarter of the mouth). At birth, the needle teeth are orientated outwards (1) and later they change to forward orientation (2). Immediately after birth, piglets fight for access to teats for reasons of survival, as those who do not succeed in securing such access are most likely to die of starvation (1, 3). The competition for teat access is most intense during the first hours of life, after which a teat preference is established during the first week (4), with a tendency for each piglet to return to the same teat for suckling [“teat order” (3)]. Piglets defend their preferred teats using their needle teeth, a phenomenon described by Fraser and Thompson (3) as “armed sibling rivalry.”

During the fights and displacements that occur to establish the teat order, piglets can inflict injuries to the faces of litter mates with their needle teeth (5). Piglets suckled more on the most anterior or posterior teats had fewer facial lesions compared to those suckle teats in the middle (5). These facial lacerations may cause facial skin necrosis forming ulcerations with brown crusts, which may extend to a large area near the mouth and closer to the eyes (6). These lesions can later open up routes for further infection and are associated with a higher prevalence of greasy pig disease (7). Zoric et al. (8) examined the prevalence of skin lesions among neonatal piglets that were teeth-resected and found that 30% of the piglets had minor facial lesions as early as 3 days of age. It is worth noting that, although teat fights and defenses are observed in piglets raised in semi-natural environments (4), the authors did not report on this. In general, there do not appear to be systematic records of the occurrence of piglet facial or sow teat lesions in such environments.

As well as injuries to littermates, competition for access to milk can lead to injuries to sows' teats although these can also be caused by external factors such as sharp edges in the environment (9). Although anatomically the teat and udder are two distinct regions and their lesions may have different causes, but due to the lack of data and mixed usage commonly found in the literature, we refer to both generally as teat lesions hereafter. Injuries to the sows' teats can make nursing uncomfortable and/or painful (2), potentially causing serious complications such as mastitis or agalactia (10), conditions whereby sows fail to produce milk (11). The teats affected by mastitis are either less functional or completely dysfunctional, thereby leading to the malnutrition of the piglets (10). When one or more teats become permanently dysfunctional (or “blind”), fostering can be used to limit the litter size to the number of remaining functional teats (see section Risk factor 4: Piglet management); ultimately, the affected sow may need to be culled (10).

In some commercial pig production systems, piglets' needle teeth are resected (reduced) to minimize facial lesions in piglets and teat lesions in sows. The two main methods to resect needle teeth are clipping and grinding (12). Clipping consists of truncation by clippers, whereas grinding is done with a rotating grindstone. Irrespective of the method, teeth resection can be total (i.e., to the gum) or partial (i.e., only the pointed tip) (13), and it can involve all piglets in a litter or be selective, meaning that the teeth of the smallest piglets in the litter are left intact. Both methods involve individual restraint of the piglet and are usually carried out without local anesthesia or analgesia presenting a specific set of animal welfare challenges, notably stress, pain, and injuries due to incorrect clipping technique and the risk of developing infections (12). Teeth resection damages the dental pulp surrounded by nerves which can be considerably painful (14). Restraint itself is also a stressor for piglets, and plasma cortisol concentration increases with restraint duration (15). It is not known what percentage of farms practices any form of teeth resection globally, but Fredriksen et al. (16) reported that it is performed in most countries in Europe. The routine application of teeth resection is prohibited by the EU legislation (17). However, it can be carried out if the farmer can demonstrate that there is a problem with sow teat lesions on the farm, with the proviso that measures are taken to prevent future lesions. Nevertheless, the causes and risk factors of such lesions are usually not investigated and, as a consequence, piglets' needle teeth are routinely resected on many farms.

We need a better understanding on the current scientific evidence on the risk factors behind piglet facial and sow teat lesions, information on the occurrence of these lesions, and the proportion of producers and other relevant stakeholders that carry out teeth resection or that use alternative methods to minimize these lesions. This is important to promote better practices and to improve pig welfare globally. Therefore, the first aim of this paper was to review the literature on the risk factors for both piglet facial and sow teat lesions, identifying possible knowledge gaps. Secondly, we collected information on the occurrence of these issues, their perceived causes and the practice of teeth resection as conducted on commercial farms by surveying pig producers and other relevant stakeholders through an online global survey. Finally, we highlighted practical alternatives to teeth resection to support management to prevent piglet facial and sow teat lesions and provided suggestions for future research on under-researched risk factors.

## Methods

### Literature review

A literature search was conducted using Google Scholar, assisted by Web of Science to cover both peer-reviewed and non-peer-reviewed gray literature. The search terms used for piglet facial lesions were: (sows OR pigs OR swine OR piglets), (face OR facial OR snout OR mouth) (lesions OR injuries OR damage OR laceration OR wound OR abrasion) in addition to each of the risk factors. Similarly, for sow teat lesions the search terms used were (sows OR pigs OR swine), (teat OR udder), (lesions OR injuries OR damage OR laceration OR wound OR abrasion) with each risk factor. A detailed list of search terms used is available in Supplementary material I.

We screened relevant literature for inclusion manually. The inclusion criteria for peer-reviewed literature were (i) either piglet facial or sow teat lesions should be included as the outcome measure, or (ii) the risk factors of either piglet facial or sow teat lesions should be discussed. For gray literature, the topic should be on observational or anecdotal description of possible risk factors causing lesions to piglets' faces or sows' teats.

The risk factors were categorized into six sections for both lesions: (a) presence or absence of teeth resection, (b) housing system, (c) litter size, (d) piglet management, (e) lack of environmental enrichment, (f) milk production and other miscellaneous risk factors.

### Teeth reduction survey

#### Survey design

An online piglet teeth reduction survey was designed in Google Forms with versions in English, Dutch, French, German, Italian, Portuguese and Spanish, and a version in Chinese was created using the online platform WJX. The survey included 20 questions in five sections (comprising 11 single-answer multiple choice, seven multiple answer multiple choice and two open-ended questions, Table 1; and full survey in Supplementary material II). The first section included one question asking whether the farm practiced teeth reduction. Those selecting “yes” to this question, went to section Methods and those selecting “no” skipped to section Results. No personal information was obtained through the survey (e.g., names, emails, IP address, etc. that could have identified individuals), and we asked for explicit consent before respondents could continue to the survey stating, “you agree that you're at least 18 years of age and the information you provided will be used for research purposes only.”

**Table 1:** A summary of the survey sections and questions for the online piglet teeth reduction survey.

**Section**	**Questions**
1. Piglet teeth reduction	Is teeth reduction practiced on the farm (yes—go to section 2 or no—skip to section 3)
2. General management practice—teeth reduction	Type of teeth reduction (clipping or grinding); how often are teeth reduced; age at teeth reduction.
3. General management practice—farrowing	Type of farrowing system; flooring in the system; training in farrowing management; piglet management strategies used; litter size; provision of nest-building materials and enrichment for piglets.
4. Problems and solutions	Why is teeth reduction practiced; the severity of piglet facial and sow teat lesions; main reported reasons for these injuries; measure(s) taken to reduce problems (multiple response options)*
5. Basic information	Location (country); size of farm; role on the farm; gender; age group.

*Respondents were provided with a pre-defined list of potential management intervention strategies/measures and asked to identify which, if any, of these strategies/measures they attempted and whether they succeeded in tackling their problems.

#### Survey distribution

Convenience sampling was used to reach as many potential respondents as possible without any specific inclusion criteria. A link to the survey was shared on social media (Twitter, LinkedIn and Facebook) and sent to colleagues *via* email. Additionally, links to the survey were distributed in the Dutch Weidemark Welfare programme and *via* the monthly Teagasc pig newsletter (with around 1,000 recipients). All respondents were involved in pig farm management at some stage during the production chain.

#### Data analyses

The survey responses to the French, German, Italian, Portuguese, Spanish, and Chinese versions were transferred into the English version of the survey in Google forms for data analysis in English. Responses were then downloaded from Google Forms into Excel and imported to R (version 4.0.5) for analysis (18). All summary statistics were obtained (using the “dplyr” package) and figures created (using the “ggplot2′ package) in R, and results were considered statistically significant at *P* < 0.05 and tendencies discussed (*P* < 0.1).

Correlations between the reported severity of piglet facial lesions with sow teat lesions were analyzed using Spearman's rank correlation (using the “spearman.test” function). Chi-square tests were used to analyse the use of enrichment, litter size, farrowing system and flooring, as well as the responses to why teeth reduction is practiced, the reported reasons for facial and teat lesions and the measures used to resolve the lesions by whether or not teeth reduction was practiced. The frequencies were cross-tabulated and analyzed using a homogeneity test for contingency tables (chi-square tests implemented in the chisq.test function in the “stats” package). Multiple answers were allowed for responses on why teeth reduction is practiced, the reported reasons for facial and teat lesions and the measures used to resolve the lesions and therefore the sample size in the Chi-square model can be larger than the number of respondents (*n* = 75).

The total number of answers selected for the following questions were summed to create numeric variables for training, management, reasons for lesions, measures that were tried and measures that worked:

i) Sow farrowing management training topics: learning about sow farrowing behavior; checking sow more regularly before farrowing; assisting farrowing; counting number of teats; feed/nutrition adjustment; checking water flowrate or consumption; and checking sow milk production
ii) Types of piglet management strategies used: cross-fostering; split suckling; use of nurse sows; artificial rearing (e.g., rescue decks); milk supplementation (*via* milk cups or similar)
iii) The main reported reasons for the occurrence of face and teat lesions: piglets' teeth are clipped or ground; piglets' teeth are NOT clipped or ground; large litter size; using conventional farrowing crates; using free farrowing pens; outdoor farrowing; flooring in the farrowing accommodation; poor milk production of sows; not enough cross-fostering; too much cross-fostering; lack of environmental enrichment/nesting material
iv) The measures tried to resolve facial and teat lesions: only used teeth reduction; avoid large litter size (i.e., keep litter size at around/below 12–13 piglets); select for sows with good mother traits; improve sow nutrition at farrowing; check on sows more frequently; increase sow water intake; provide early supplementary piglet nutrition; frequent cross-fostering; split suckling; use nurse sows; artificial rearing; provide nesting material/enrichment in the farrowing crate/pen)
v) The measures reported to have worked to resolve the lesions (same options as previous)

These new numeric variables were analyzed for differences between those who practice teeth reduction or not and by the reported severity of facial and teat lesions using non-parametric tests for group differences (the “wilcox.test” function for teeth reduction and the “kruskal.test” function for the severity). Finally, the non-parametric Kruskal Wallis test (using the “kruskal.test” function) was conducted with the reported severity of piglet facial and sow teat lesions as the dependent variables with the following as explanatory variables: litter size, farrowing system, floor type and material, the use of enrichment, farm size and whether or not teeth reduction is practiced. *P*-values were corrected for multiple testing using the Bonferroni correction.

## Results

### Overview of literature search results

In total, 51 articles matched the search criteria (41 peer-review journal articles and 10 pieces of non-peer-reviewed gray literature). The majority of research focussed on the effects of the presence or absence of teeth resection, different methods of teeth resection, followed by the housing systems (conventional farrowing crate vs. free farrowing pens) (Table 2). Some studies investigated the risk factors of litter size and piglet-related management strategies but very few studies looked into the effects of enriching the environment or of milk production on the occurrence of these lesions. These were the six major risk factors identified (Table 2). In the following sections, each risk factor is discussed separately for both piglet facial and sow teat lesions.

**Table 2:** The number of records found on each factor for piglet facial and sow teat lesions, or both.

**Lesion**	**Teeth resection**	**Housing**	**Litter size**	**Piglet management**	**Environmental enrichment**	**Milk production**	**Other factors**
Piglet facial	8	3	3	7	0	3^†^	1
Sow teat	3	12	3	1	0	0	0
Both	8	1	5	3	2	0	1
Total	19	16	11	11	2	3	2

† Including research on the indirect effect of milk supplementation.

Some records discussed multiple factors and therefore the total number 63 is greater than the number of records found (*N* = 51).

### Overview of teeth reduction survey results

Seventy-five respondents from 17 countries answered the survey providing valid responses. Although somewhat limited in the total quantity of responses, detailed demographics of the respondents including respondents' country, age, gender and role on the farm, and the farm size are provided in Supplementary material III, Table 1. In summary, 64.0% (*n* = 48) of respondents were based in Europe, 66.7% (*n* = 50) were male, 38.7% (*n* = 29) were farm owners and 48.0% (*n* = 36) worked on medium-sized farms (100–999 sows). Of note, while attempts were made to engage pig industry participants from across the globe, two large segments of world pig production (USA, no respondents; China, three respondents) are not proportionately represented; therefore, the authors acknowledge that findings may not be indicative of production characteristics and efficacy of strategies outside of the countries more heavily represented.

#### Reported occurrence of piglet facial and sow teat lesions

Respondents were asked to indicate the severity of piglet facial and sow teat lesions on their farms (Table 3). More than half of the respondents indicated that such lesions are manageable without needing to change management practices (teat: 38, 50.7%, face: 39, 52.0%), with some reporting that they never encountered these lesions (teat: 17, 22.7%, face: 18, 24.0%) or that they needed to adjust management practices to keep it manageable (teat: 18, 24.0%, face: 16, 21.3%). Only few respondents found the problems unmanageable (teat: 2, 2.6%, face: 2, 2.7%). The reported severities of piglet facial and sow teat lesions were correlated [Spearman's rank correlation (r_s_) = 0.727, *P* < 0.001; Table 3].

**Table 3:** Correlation between the reported severity of piglet face and sow udder lesions [Spearman's rank correlation (r_s_) = 0.727, *P* < 0.001].

**Severity**		**Sow teat lesions**			
		**Never**	**Manageable without needing to change management practices**	**Needed to adjust management practices to keep it manageable**	**Not manageable**
Piglet face lesions	Never	14 (18.7%)	4 (5.3%)	0 (0.0%)	0 (0.0%)
	Manageable without needing to change management practices	2 (2.7%)	30 (40.0%)	7 (9.3%)	0 (0.0%)
	Needed to adjust management practices to keep it manageable	1 (1.3%)	4 (5.3%)	11 (14.7%)	0 (0.0%)
	Not manageable	0 (0.0%)	0 (0.0%)	0 (0.0%)	2 (2.7%)

#### Reported reasons for piglet facial and sow teat lesions

**Figure 1:**
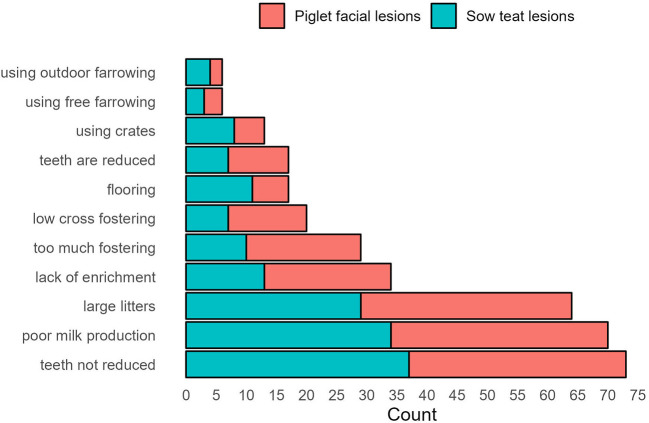
Counts of responses to the question: *What do you think are the main reasons for the occurrence of these problems (piglet facial and sow teat lesions)?* The question included the option to select from a pre-defined listing of reasons as shown on the vertical axis. Counts of these reasons selected for piglet facial lesions (in red) and sow teat lesions (in blue) are shown from most (at the bottom) to least (at the top) selected.

The main reasons reported for the occurrence of piglet facial lesions, sow teat lesions or both lesion types are shown in Figure 1. The top three reported risk factors for both lesion types included “teeth not being reduced,” “poor milk production,” and “large litters”. Further breakdown of the reported reasons by the reported severity for the occurrence of piglet facial and sow teat lesions are shown in Supplementary material III, Table 2.

#### Reported measures to prevent piglet facial and sow teat lesions

**Figure 2:**
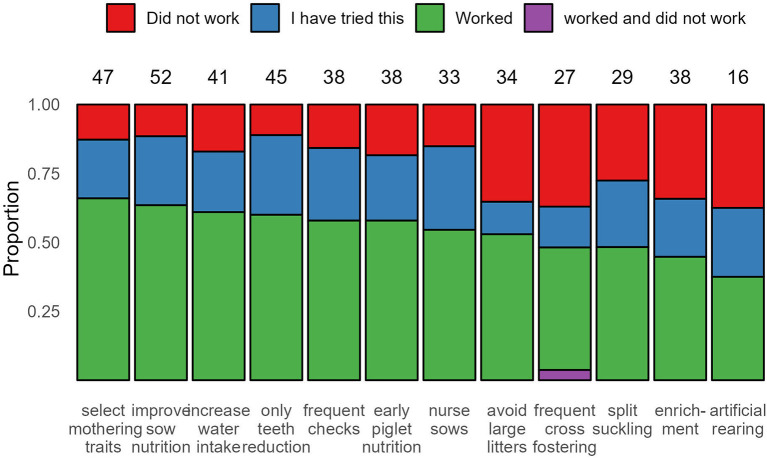
Proportion of response selections (“Did not work”, “I have tried this”, “Worked” or “Worked and did not work”) to the question: *What measures have you tried other than teeth reduction to solve your problems, and which ones worked/did not work?* Counts of the total number of respondents who selected each measure (including “I have tried this,” “Did not work” and “Worked”) is shown above the bars.

When asked what intervention measures were tried to solve the issues of piglet facial and sow teat lesions, as well as whether the measures worked or not, respondents showed a range of responses (Figure 2).

A more detailed breakdown of the survey results will be presented hereafter in accordance with each risk factor.

### Risk factor 1: Presence or absence of teeth resection

#### Literature on piglet facial lesions

Teeth resection was the subject of most of the retrieved scientific studies related to the occurrence of piglet facial lesions. As early as 1975, Fraser (5) investigated the function of the needle teeth and the effect of teeth clipping on the lesions. The author found that facial lesions occurred mainly in unclipped litters, and that the majority of teeth-clipped litters recorded no facial lesions at all (42 out of 50, cf. 14 out of 42 for unclipped litters with no lesions). However, teeth status did not affect the frequency of fighting or growth, nor the stabilization of teat order. Unclipped litters with a smaller litter size had fewer facial lesions than those with a larger litter size, which suggested the interaction between teeth status and litter size (more discussion in section Risk factor 3: Litter size).

Most studies reported that intact teeth increased the risk of facial lesions [on day 7, day 14 and day 21.5–22 after birth (19); on days 3 and 13 after birth (20); day 4-6 after birth (21); days 7 and 21 after birth but only on one study farm (22); most days before weaning (23), but Hansson and Lundheim (24) found that piglets with intact teeth had similar facial lesion scores as the ones with ground needle teeth. Similarly, Fu et al. (25) also found no difference in piglet front (face to back shoulder) lesion scores between teeth-clipped and intact piglets, on days 2, 5, 7, 10, 15, and 21 after birth. Some studies found the difference in lesions was transient and no longer present at weaning (21). Furthermore, Brown et al. (19) found most lesions recorded were superficial without infection, and they had no effect on mortality and weight gain, which agreed with Bates et al. (20).

When selective teeth clipping was practiced (i.e., leaving the needle teeth of the smallest piglets in a litter unclipped), only a small percentage of facial lesions was reported (26). Therefore, in an industry report, this practice was recommended to producers who may want to phase out teeth resection (7). Weary and Fraser (27) compared piglets with fully-clipped (removing the needle teeth above the gum line), partially-clipped (removing one-third of the needle teeth) and intact teeth using a within-litter comparison method (i.e., in each litter, one of the three treatments was applied to the right side of the mouth and another to the left side of the mouth for all piglets within that litter). The authors always found more facial lesions on the side of a piglet's face when it was facing against the intact teeth side of another piglet, and several litters with intact teeth from one side needed to be removed from the study due to a higher frequency of severe facial lesions.

Although teeth resection can decrease the risk of piglet facial lesions, it is worth noting that the procedure itself can cause significant lesions in the piglets' mouth and on their teeth (28, 29). It is recognized that teeth resection, especially when performed in an unskilled manner, can cause gum injuries and create a route for infection along the gum line and into the bloodstream (30, 31).

In gray literature, a consensus continues to exist in the veterinary and producer advisory circles that teeth clipping should be practiced in order to reduce lacerations on piglets' faces (22, 31–33) and there can be complications from facial lesions such as greasy pig disease caused by bacterial infection on the skin (7). Wilkinson and Blackshaw (34) found no difference in the interactions between teeth clipping or not, and small or large litters, but reported anecdotally that six out of 40 unclipped litters had bad facial lesions. However, Estienne et al. (22) showed that there was variation between farms and the effect of teeth clipping on the piglet facial lesions.

#### Literature on sow teat lesions

Fu et al. (25) recorded higher teat lesion scores (anterior, middle and posterior) in sows nursing non-teeth-clipped piglets compared to clipped ones. Estienne et al. (22) reported that teeth clipping reduced sow teat lesions on day 7, but not on day 21 post-farrowing. Other studies found no difference in the level of teat damage between intact, ground or clipped litters (21, 24), or only a tendency of fewer sows with teat lesions with clipped litters (28). Gallois et al. (29) reported a slight increase in mild lesions in sows' anterior teats only on day 8 post-partum when piglets' teeth were kept intact compared to teeth-clipped or ground piglets. Similar to the outcome for piglet facial lesions, when practicing selective teeth clipping, only minimal cases of sow teat injuries were reported (26).

By contrast, a Swedish study showed that either clipping or grinding piglets' needle teeth increased the risk of mastitis in sows, which could be due to bacteria introduced by infection in the pulp cavity following teeth resection procedures (35), although the cause and effect is unclear.

Similar to piglet facial lesions, the advisory resources to producers from gray literature commonly recognized that teat lesions can be prevented by resecting piglets' teeth (11, 22, 32, 36), as these injuries are considered a potential cause for mastitis and agalactia in sows (11).

#### Survey results

Contrary to the focus found in the literature, just over half (38, 50.7%) of the respondents indicated that they did not practice teeth reduction on their farm, and just under half (37, 49.3%) practiced the procedure. For those performing teeth reduction, the majority only used grinding (22, 59.5%), while some used clipping (13, 35.1%) and two respondents reported using both methods (5.4%). Almost all performed the procedure within 48 h after piglets were born (31, 83.8%), while five performed it between piglets' age of daya 3–7 and one did it *ad-hoc* whenever they see a problem occurring. Thirteen (35.1%) individuals reported performing the procedure on all litters and all piglets, fourteen (37.8%) reported reducing the teeth of all litters but avoiding the small piglets, five (13.5%) reduced the teeth of most litters and all piglets within a litter, and a small number (3, 8.2%) reported resection on most litters but avoiding small piglets. Two (5.4%) respondents occasionally perform teeth reduction when they see a problem.

There was a difference in the reported severity of piglet facial lesions (effect size = 12.785, *P* < 0.001) between those who practice teeth reduction and those who do not, as fewer respondents who practice teeth reduction never saw piglet facial lesions, whereas more respondents who practice teeth reduction reported that they needed to adjust management practices to keep lesions manageable (Supplementary material III, Table 3).

The reported reasons why respondents practice or used to practice teeth reduction, or their opinion on why this procedure is performed, are shown in Table 4. The top two reasons speculated for carrying out (or not) the procedure were problems with piglet facial and sow teat lesions. When given the option “other”, respondents reported that they simply do not perform the procedure or that the procedure is performed to reduce piglet mortality or morbidity, which included: “*More piglet loss due to restless sows (15%), was proven by research*” and “*Dramatic increase in Greasy Pig disease especially in gilt litters*”. Including only the main options (not the “other” options) for why the procedure is performed, there was no difference between the reasons reported by those who practice or do not practice teeth reduction.

**Table 4:** Reported reasons why teeth reduction is performed by respondents answering “No” or “Yes” to current use of teeth reduction on their farm.

**Why do you practise teeth reduction (or what are reasons based on past experience/opinion)? (Multiple answers possible)**	**Teeth reduction**		
	**No (*n* = 38)**	**Yes (*n* = 37)**	**All data (*n* = 75)**
Problems with piglet facial lesions	24 (64.9%)	27 (71.5%)	51 (68.0%)
Problems with sow teat injuries	17 (45.9%)	26 (68.4%)	43 (57.3%)
Standard procedure	9 (24.3%)	11 (28.9%)	20 (26.7%)
Ease of overall management	0 (0.0%)	6 (15.8%)	6 (8.0%)
Insufficient milk production	3 (8.1%)	3 (7.9%)	6 (8.0%)
Too large litter sizes	2 (5.4%)	2 (5.3%)	4 (5.3%)
Other “do not perform”	9 (24.3%)	0 (0.0%)	9 (12.0%)
Other “piglet morbidity/mortality”	1 (2.7%)	4 (10.5%)	5 (6.7%)

Multiple answers were possible; therefore, *N* = 130 for total answers recorded, which is larger than the number of respondents (*n* = 75).

**Figure 3:**
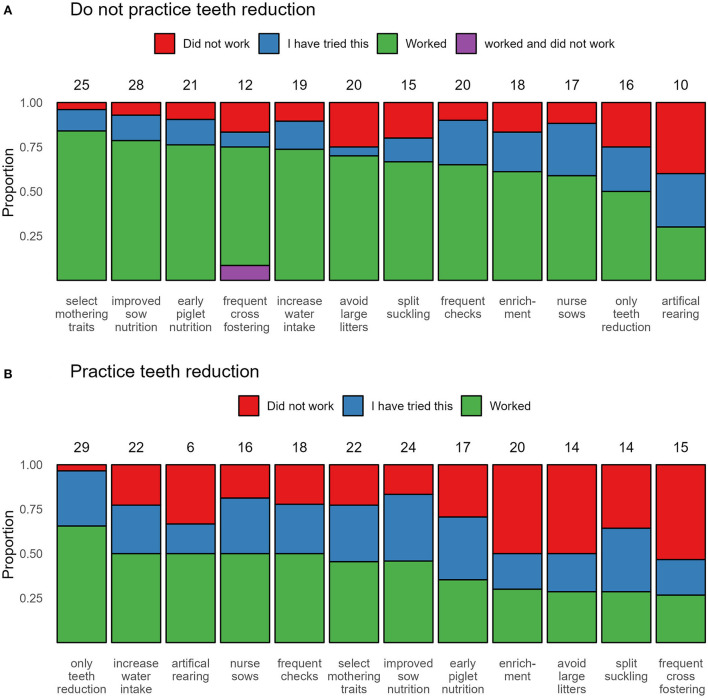
Reported measures to prevent piglet facial and sow teat lesions varied between the two groups of respondents (with or without performing teeth reduction). Counts of the total number of respondents who tried each measure are shown above the bars. **(A)** Do not practice teeth reduction. **(B)** Practice teeth reduction.

Similarly, there was no difference in the respondents' opinions about the causes of piglet facial and sow teat lesions between those who practice teeth reduction and those who do not. However, those who do not practice teeth reduction considered “large litters” as the top cause, whereas those who practice teeth reduction considered “teeth not reduced” as the main cause of the lesions (Supplementary material III, Figure 1). The reported measures that they employed to solve the issues of piglet facial and sow teat lesions, as well as whether they worked or not are shown in Figure 3.

The total number of answers selected by respondents for (1) training staff on sow management at farrowing, (2) piglet management strategies used during farrowing, (3) reasons for the presence of piglet facial and sow teat lesions, and (4) the measures that were tried to resolve these lesions, did not differ between those who practice teeth reduction and those who do not (Supplementary material III, Table 4).

In summary, the literature review indicated a positive effect of teeth resection to control piglet facial and sow teat lesions, although some studies found the difference in lesions was most apparent in the initial days during suckling. The gray literature almost unanimously indicates that teeth resection is the necessary procedure to prevent piglet facial and sow teat lesions. The survey showed that only about half of the respondents use teeth reduction to control these lesions. The respondents that do resect piglets' needle teeth considered the issues of lesions more severe. They consider them only controllable by teeth reduction, which is why they were still practicing it, but this is only numerically different.

### Risk factor 2: Housing system

The risk factors for piglet facial lesions and sow teat lesions associated with housing system include farrowing system and flooring. These factors can be the direct cause of such injuries or may indirectly influence teat order fights of piglets.

#### Literature on piglet facial lesions

There are indications of an effect of the suckling environment on piglet facial lesions (37). In a study by Lohmeier et al. (37), piglets born to sows housed in farrowing crates had more facial lesions than piglets born to sows in farrowing pens. This was purportedly because the duration of suckling events was longer in farrowing pens, and penned sows were therefore less likely to terminate suckling events. However, Hemsworth et al. (38) only found a difference between crates and pens, with more total skin injuries (86% of which were on the ears, face, shoulders and neck) in crated piglets, but only in one of two studies reported in the paper. Group pen vs. single pen lactation housing can also reduce facial lesions in piglets, with sows and piglets mixing at 6 days post-partum (39). Flooring type within the farrowing system may also impact lesion scores. Zoric et al. (40) compared three farrowing pens of similar size, one with solid floor and chopped straw, one with part slatted floor and chopped straw and one with deep peat floor. At 3 days post-partum, piglet facial lesions were higher in the peat pen system compared with the other two systems, but the difference had disappeared by day 10.

#### Literature on sow teat lesions

Compared to literature on piglet facial lesions, there are more studies that examined the impacts of housing on sow teat and udder injuries and there appears to be consensus that systems with increased space or freedom of movement for the sow, offer protective effects. This is presumably by enabling the sow to protect her udder from unwanted attention from her piglets. Sows housed in conventional crates compared with crates that open (41) or individual farrowing pens (37, 42) had more teat and udder skin lesions, respectively. The length of time of confinement within a crate can also impact lesions, with sows temporarily crated from day −1 to +4 post-partum having markedly higher chances of teat lesions than sows not crated and sows either crated from day 0 to +3 or day −1 to +6 (43).

For sows that are never crated during lactation, space allowance may have an effect. Koller et al. (44) compared pen sizes of 7.6, 4.9, and 4.1 m^2^ and found that sows in the two smaller pen sizes showed significantly more lesions on udder and teats than sows in the largest pen size. Studies comparing single pen lactation with group lactation (i.e., more than one sow and litter farrowing in a common area with mutual interactions between litters) found fewer teat and udder lesions in the group housed sows (35, 39). In line with this, Brown et al. (19) recorded less sow teat damage in outdoor herds even when piglets' teeth were left intact.

A few studies also examined the flooring type and its impact on udder lesions. Norring et al. (45) found no treatment effect on udder lesions when comparing bare concrete flooring with polyurethane-coated concrete. Similarly, Bonde et al. (46) compared 10 farms which had crates on either fully- or part-slatted floors, and with the slats either being metal, plastic, concrete, cast iron or triangular profile, and found no effect of type on udder lesions which ranged in incidence from 2 to 12% (mean 8%) of sows. They noted that lesions appeared to be primarily caused by the sows' own hooves when slipping during posture changing, which may be due to poor floor quality. A study comparing solid, straw bedded floors with fully- or part-slatted perforated metal or plastic-coated woven wire showed no teat damage at weaning on 95% of sows bedded on straw, but no teat damage on only 54–62% of sows housed on the slatted systems (9).

The type of sow housing during the gestation period may also influence the occurrence of teat lesions. Anil et al. (47) found numerically more teat lesions (not statistically significant) in sows housed in group pens with electronic sow feeders (40–60 sows per group) compared to housing individually in stalls. This may be partly due to the freedom of movement and the possibility of coming across sharp edges, being stepped or laid upon while resting in the surroundings for the group-housed sows.

#### Survey results

The farrowing system and flooring used on the farms of the survey respondents is shown in Table 5. The majority of the respondents used conventional farrowing crates on fully or partly slatted floors. The reported severity of piglet face and sow teat lesions differed by farrowing system (face: effect size = 8.938, *P* < 0.05, teat: effect size = 9.705, *P* < 0.05) and flooring type (face: effect size = 10.623, *P* < 0.05, teat: effect size = 10.346, *P* ≤ 0.05) as more of those with alternative farrowing systems never see these lesions or reported they were manageable without changing management practices (Table 6).

**Table 5:** Counts of farrowing system, flooring and use of enrichment reported by respondents.

**System**	**Flooring^a^**				
	**Fully slatted**	**Partly slatted**	**Fully or partly slatted**	**Solid**	**Unknown**
Conventional crates (*n* = 63)	27	24	1	0	11
Free farrowing pens (*n* = 7)	2	2	0	1	2
Outdoor farrowing (*n* = 5)	0	0	0	5	0

a Totals for floor surface type are as follows: fully slatted (*n* = 29), partly slatted (*n* = 26), fully or part slatted mixture (*n* = 1), solid floor (*n* = 6), unknown (*n* = 13).

**Table 6:** The reported severity of sow teat (teat) and piglet facial (face) lesions according to the farrowing system and floor type in the farrowing accommodation.

**Severity**	**Farrowing system**		
	**Conventional farrowing crates**	**Free farrowing pens**	**Outdoor farrowing**
Teat: Never	10 (15.8%)	3 (42.9%)	4 (80.0%)
Teat: Manageable without changing practices	34 (54.0%)	3 (42.9%)	1 (20.0%)
Teat: Needed to adjust management practices	17 (27.0%)	1 (14.2%)	0 (0.0%)
Teat: Not manageable	2 (3.2%)	0 (0.0%)	0 (0.0%)
Face: Never	11 (17.5%)	3 (42.9%)	4 (80.0%)
Face: Manageable without changing practices	35 (55.5%)	3 (42.9%)	1 (20.0%)
Face: Needed to adjust management practices	15 (23.8%)	1 (14.2%)	0 (0.0%)
Face: Not manageable	2 (3.2%)	0 (0.0%)	0 (0.0%)
	**Flooring**		
	**Fully slatted**	**Partly slatted**	**Solid**
Teat: Never	4 (13.8%)	4 (15.4%)	5 (83.3%)
Teat: Manageable without changing practices	16 (55.2%)	14 (53.9%)	1 (16.7%)
Teat: Needed to adjust management practices	8 (27.6%)	7 (26.9%)	0 (0.0%)
Teat: Not manageable	1 (3.4%)	1 (3.8%)	0 (0.0%)
Face: Never	4 (13.8%)	7 (26.9%)	5 (83.3%)
Face: Manageable without changing practices	15 (51.7%)	14 (53.9%)	1 (16.7%)
Face: Needed to adjust management practices	9 (31.1%)	4 (15.4%)	0 (0.0%)
Face: Not manageable	1 (3.4%)	1 (3.8%)	0 (0.0%)

Some respondents reported flooring as a reason for the presence of sow teat (14.7%) and piglet facial (8%) lesions, and a few reported using specific farrowing systems as a reason, with crates (teat: 10.7%; face: 6.7%) reported more often than free farrowing pens (teat: 4.0%; face: 4.0%) and outdoor farrowing (teat: 5.3%; face: 2.7%) (Supplementary material III, Table 2). Although most respondents used conventional farrowing crates, numerically more of those using alternative farrowing systems (i.e., free farrowing pens and outdoor farrowing) did not practise teeth reduction (Supplementary material III, Table 5).

In summary, literature shows that farrowing systems which give sows more freedom of movement and more space may reduce the risk of sow teat lesions, as sows have more control over nursing, whereas the effect of flooring material is less clear. In accordance with the literature review, the survey also showed that the issue of lesions was less severe on farms not using conventional farrowing crates, and numerically fewer respondents reported practising teeth reduction in alternative farrowing systems.

### Risk factor 3: Litter size

#### Literature on piglet facial lesions

The teat order is established as soon as possible after birth. As failure to secure a teat may result in the unsuccessful piglets potentially starving to death, competition for teat access occurs even if the number of functional teats is sufficient for all littermates (2). In large litters [14–20 piglets according to (13)] the incidence of fights is higher (5, 12, 13, 24, 34, 42, 48) than in smaller litters. The studies that actually measured piglet facial lesions found that these occur more frequently in larger litters compared to smaller ones (5, 13, 24, 42). This is an indication that litter size influences both the proportion of “teat fighters” and the prevalence of the resulting injuries (42), as piglets fight for teat access by pushing away other piglets trying to nurse from the same teat or by biting them with their needle teeth.

#### Literature on sow teat lesions

Some studies show that litter size influences the number and severity of teat wounds in sows. The risk of having at least one teat wound and the number of teat wounds per sow increased significantly with an increase in litter size at weaning (49). This was also confirmed by Norring et al. (45), who found that the litter size affected the change in the proportion of wounded teats: sows with bigger litters had more wounded teats and that sows with bigger litters had more wounded teats. However, Kobek-Kjeldager et al. (42) did not find an effect of litter size on udder or teat abrasions but only an effect of time when litter size was accounted for. As opposed to fighting among piglets, which tended to decrease from day 7 onwards concurrently with the proportion of associated facial lesions, udder abrasions in sows increased over the course of lactation, affecting 85% of sows on day 28, whereas teat abrasions remained relatively stable, affecting 14% of sows on day 28 (42). These results are mostly in line with previous conclusions reviewed by Rutherford et al. (50), who classified the evidence linking litter size with udder damage to the sow as being “sound”, with a high certainty of negative welfare impact. A possible explanation is that in larger litters piglets can fail to establish a stable teat order and therefore the incidence of teat fighting, missed suckling, udder massage and udder damage may increase over time (42).

#### Survey results

The average litter size (born alive) in the responding farms is shown in Table 7. The reported severity of piglet face and sow teat lesions did not differ by litter size (data not shown), but numerically litter size was different between practices of teeth reduction or not, with more of those practicing teeth reduction having larger litters than those who do not resect teeth.

**Table 7:** Reported litter size according to whether or not teeth reduction is currently practiced.

**What is your average**	**Teeth reduced?**		
**litter size (born alive)?**	**No**	**Yes**	**All**
<piglets	2 (2.7%)	0	2 (2.7%)
11–12 piglets	8 (10.7%)	3 (4.0%)	11 (14.7%)
13–15 piglets	23 (30.7%)	21 (28.0%)	44 (58.7%)
16–18 piglets	5 (6.7%)	12 (16.0%)	17 (22.7%)
More than 18 piglets	0	1 (1.2%)	1 (1.2%)

When asked to select the reasons for the occurrence of piglet face and sow teat lesions, “large litters” was the third most selected option, after “teeth not being reduced” and “poor milk production”, as 46.7 and 38.7% selected this option for piglet facial and sow teat lesions, respectively (Supplementary material III, Table 2). For those who did not practice teeth reduction, “large litters” was the top reported reasons for piglet facial and sow teat lesions (Supplementary material III, Figure 1). In contrast, too large litter size was selected as the least frequent reason to carry out teeth reduction (Table 4).

Thirty-four respondents reported “avoiding large litters (keep litter size at around/below 12–13 piglets)” as a measure that they employed to tackle problems with piglet face and sow teat lesions (Figure 2). Of those, 18 (52.9%) reported that it worked, and 14 out of those did not practice teeth reduction.

In summary, there is some evidence that large litter sizes increase the incidence of fighting to establish teat order and also the risk for piglet facial lesions. Additionally, sows with larger litters have more teat wounds, which can negatively affect sow welfare due to acute or chronic mastitis. This agrees with the survey results, with many respondents acknowledging large litters as a risk factor for these lesions and one of the top-rated measures taken to alleviate these issues, especially for those who do not practise teeth reduction.

### Risk factor 4: Piglet management

#### Literature on piglet facial lesions

Cross-fostering was traditionally performed to improve the survivability chances of low-birth-weight piglets (31) but is increasingly used to manage large litters (13). Given that the practice involves the transfer of piglets into unfamiliar litters, it is also associated with aggression between unfamiliar piglets as they fight for access to teats (51). Hence, cross-fostered piglets typically have more facial lesions than resident piglets as resident piglets defend “their” teats (19, 52–54). However, there is also contradictory evidence suggesting that in litters with cross-fostering, facial lesion scores tended to be lower than in litters without cross-fostering (24). Nevertheless, there is evidence that when cross-fostering is improperly practiced on farms such that piglets are cross-fostered late or numerous times, this could exacerbate associated animal welfare problems (55), not limited to increased lesions.

Nurse sow strategies are also applied to manage large litters. A nurse sow is a lactating sow given younger foster piglets, when her own piglets are transferred to another sow or weaned. Two main types of strategies are used (13); the nurse sow is given new-born piglets when her own piglets are weaned (the one-step strategy), or the nurse sow is given 1-week-old piglets when her own older piglets are weaned or transferred to another sow (the two-step strategy). Although this practice essentially also involves cross-fostering, Sørensen et al. (56) found no effect of different nurse sow strategies on facial lesions in piglets.

Early socialisation can be achieved by exposing piglets to unfamiliar piglets in other litters between conventional farrowing crates while still suckling their dam (57). This is a valuable strategy to reduce aggression and the associated stress at weaning (58). While some studies (58, 59) reported an increase in piglet lesions specifically to the front of the body, associated with early socialisation, they did not distinguish the facial area. More specifically, Camerlink et al. (60) reported a small increase in the number of piglets with facial wounds after socialisation (between two litters) pre-weaning; however, a recent study found no such effect when piglets were co-mingled across 3 litters pre-weaning (61).

#### Literature on sow teat lesions

Schmitt et al. (62) found a tendency for udder lesions to increase in sows that farrowed large litters and were thereafter left with an equalised litter of 12 piglets (a mix of own and fostered piglets) after transferring of heavier piglets to nurse sows, when compared to sows used in three nurse sow treatment strategies or to sows that reared all their own piglets (in a litter of 12). These findings contrast with Sorensen et al. (56) who reported a significantly higher risk of udder lesions in nurse sows in conjunction with more teat fights in nurse sow litters compared to non-nurse sow litters.

Similar to the results on piglet facial lesions, Camerlink et al. (60) found that sows whose piglets were socialised at 10 days of age (by mixing with other litters while still suckling their dam) had more teat damage than sows whose piglets were not socialised indicating there was some fighting at the udder between resident and alien piglets. On the contrary, Van Kerschaver et al. (61) did not find early socialisation affected udder or teat lesions.

#### Survey results

Respondents reported that they use or have used several management strategies on suckling piglets. The most common were the use of cross-fostering (49, 65.3%), nurse sows (49, 65.3%), split suckling (41, 54.7%) and milk supplementation (31, 41.3%). Fewer respondents reported using artificial rearing (12, 16.0%) and some reported using none of the listed management strategies (10, 13.3%). The use of piglet management strategies did not differ by whether or not teeth reduction was used (data not shown).

When asked to select the reasons for the occurrence of piglet facial and sow teat lesions, too much cross-fostering (face: 19, 25.3%; teat: 10, 13.3%), and low cross-fostering (face: 13, 17.3%; teat: 7, 9.3%) were both selected by more respondents for piglet facial lesions than sow teat lesions.

The measures related to management strategies reported to solve the issue of piglet facial and sow teat lesions included early piglet nutrition (tried: 38; worked: 22, 57.9%), using nurse sows (tried: 33; worked: 18, 54.5%), split suckling (tried: 29; worked: 14, 48.3%), frequent cross-fostering (tried: 27; worked: 12, 44.4%) and artificial rearing (tried: 16; worked: 6, 37.5%) (Figure 2).

In summary, most literature studies reviewed showed that cross-fostering increased piglet facial lesions but were less conclusive for facial lesions across nurse sow strategies presented in the literature. Early socialisation is beneficial for piglets to reduce aggression and stress later on but may increase the risk of piglet facial and sow teat lesions. The survey respondents indicated many management strategies to help alleviate these lesions, especially the provision of early piglet nutrition supplementation and cross-fostering, but also expressed ambivalence in terms of how much cross-fostering would be beneficial.

### Risk factor 5: Lack of environmental enrichment

#### Literature on piglet facial and sow teat lesions

The literature on how environmental enrichment influences piglet facial or sow teat lesions is sparse. Most relevant studies reported on the possible indirect influences, e.g., *via* the sow and the farrowing system, as well as how the sow is managed (see sections Risk factor 2: Housing system and Risk factor 4: Piglet management).

Very few studies focussed on effects of varying the piglets' environment on facial or teat lesions. Early work (63) found that an impoverished environment can channel the exploratory behaviour of piglets into the massaging or nibbling of littermates or the udder of the sow. Within the range of exploratory behaviours, chewing substrates increased when straw was available, but in a barren crate environment, this was redirected to massaging or nibbling the sow or littermates, as well as manipulating the udder of the sow. Furthermore, in the impoverished environment, piglets were also more restless due to the exploratory behaviours directed toward each other. When a creep feeder was introduced, this reduced massaging and nibbling littermates and the sow (63). This suggests that an enriched environment in early life could assist with reducing piglet facial and sow teat lesions.

Building on these findings, Lewis et al. (64) evaluated the effect of providing shredded paper or rope, as potentially enriching substrates to piglets and sows in conventional farrowing crates. The study found some positive effects of providing enrichment on reducing both facial and teat lesions; however, both effects were weak. Piglets (with clipped teeth) in litters that were offered shredded paper tended to have fewer facial lesions. There was a numerical difference in the pre-weaning udder lesion scores on certain days, with sows from barren crates having more teat lesions (day 27), although the scores did not differ overall between treatments. This suggests that there may be a positive effect of enrichment, where piglets are directed away from the sow and each other, potentially decreasing the amount of manipulating and nibbling behaviours.

#### Survey results

The respondents' use of nesting materials and environmental enrichment is reported in Table 8. The reported use of nest-building materials or enrichment was similar between those practising teeth reduction or not (data not shown), and the reported severity of piglet face and sow teat lesions did not differ with the use of enrichment (data not shown).

**Table 8:** Counts of use of enrichment by farrowing systems as reported by respondents.

**System**	**Enrichment**				
	**Both nesting materials and piglet enrichment** **(*n* = 24)**	**Nesting materials only** **(*n* = 14)**	**Piglet enrichment only** **(*n* = 13)**	**None** **(*n* = 23)**	**Other** **(*n* = 1)**
Conventional crates	6	4	5	12	0
(*n* = 63)	9	7	3	4	1
	1	0	0	0	0
	2	0	4	5	0
Free farrowing pens	1	0	0	1	0
(*n* = 7)	1	0	1	0	0
	1	0	0	0	0
	0	2	0	0	0
Outdoor farrowing	3	1	0	1	0
(*n* = 5)					

Several respondents perceived the lack of enrichment as a reason for the presence of piglet face (28.0%) and sow teat (17.3%) lesions, which was the fourth highest reason reported, after teeth not being reduced, poor milk production and larger litters (Supplementary material III, Table 2). Thirty-eight respondents reported using enrichment as a measure to solve the issue of piglet facial and sow teat lesions (Figure 2). Of those, 17 (44.7%) reported that it worked, and 11 of those did not practice teeth reduction.

In summary, early work found a benefit of providing piglets with opportunities to chew so that they did not redirect this behaviour to littermates or the sow; however, this does not appear to be studied (or reported) in later investigations on piglet facial and sow teat lesions. Interestingly, several survey respondents applied the use of enrichment to manage these lesions and that several of those reported that this works. This warrants further exploration also with a view to provide adequate environmental enrichment for pigs, which is essential to improve their welfare in general (65).

### Risk factor 6: Milk production

#### Literature on piglet facial and sow teat lesions

Anecdotal evidence suggests that poor milk production causes hungry and distressed piglets to fight for access to a teat and thereby potentially causes not only facial lesions but also injuries to the sows' teats. The vocalisations of the fighting piglets further disturb the sows, causing them to lie on their bellies or to dog sit (66) and thereby limit access to the udder. Hence the teat order is disrupted, and piglets must fight to re-establish it (67), exacerbating the problem. Rydhmer et al. (68) found that the frequency of disturbed milk production in sows varied from 0.4 to 35% between herds. Furthermore, Fraser (69) reported an average of 27.2% milk let-down failures in sows during nursing. Nevertheless, there were no published studies on the link between milk production or maternal behaviour and the incidence of either piglet facial or sow teat lesions. However, studies involving artificially fed piglets shed some light on the link between milk intake or hunger in piglets and piglet facial lesions. Kobek-Kjeldager et al. (42) found a positive effect of access to milk replacer on piglet snout abrasions compared to piglets without access to milk replacer but no effect on the amount of fighting for access to a teat. Similarly, Pustal et al. also found more piglets that needed to be treated from facial lesions when they were not provided with milk supplementation (70). In contrast, Schmitt et al. (71) found no difference in snout lesions between dam and artificially reared piglets.

#### Survey results

“Poor milk production” was almost equally reported as the top reason for the presence of piglet facial and sow teat lesions (face: 36, 48.0%; teat: 34, 45.3%), with reported percentages nearly equal to those that reported “teeth not reduced” (face: 36, 48.0%; teat: 37, 49.3%). However, when asked the rationale for teeth reduction, only six respondents (8.0%, Table 4) selected “Insufficient milk production.” For the measures reported to potentially solve the issue of piglet facial and sow teat lesions (Figure 2), options related to milk production were frequently tried and worked, including: selecting mothering traits (tried: 47; worked: 31, 66.0%), improve sow nutrition (tried: 52; worked: 33, 63.5%), increase water intake (tried: 41; worked: 25, 61.0%) and frequent checks (tried: 38; worked: 22, 57.9%). How frequently each measure was considered successful or not between those that did or did not practice teeth reduction is shown in Figure 3.

**Figure 4:**
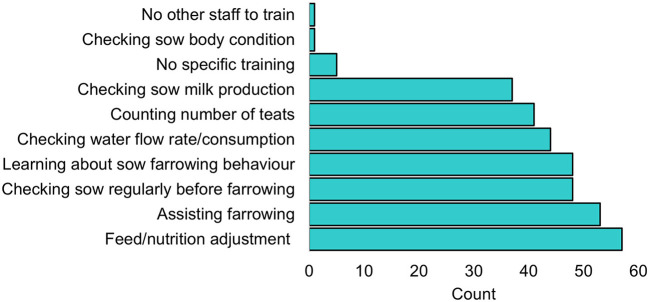
Frequency of the reported selection of sow management training topics for farm staff.

In terms of the training of staff on farrowing management, respondents selected a median of 5 (mean: 4.39, min: 0, max: 7) training topics. The frequency of the training options selected is shown in Figure 4. Many of the highly rated training topics were related to improving sow milk production. Farrowing management training did not differ by whether or not teeth reduction was practiced (data not shown).

In summary, poor milk production was recognized as an important risk factor behind piglet facial and sow teat lesions by the respondents who expressed the necessity to monitor and maintain sow milk production to prevent the lesions. However, we could not find scientific literature on this subject, and only few studies indirectly showed evidence that milk supplementation for piglets could reduce piglet facial lesions.

### Other miscellaneous risk factors

Poor health in sows and their piglets affects sow teat and piglet facial lesions through several indirect routes such as those relating to milk production outlined in section Risk factor 6: Milk production. However, pathogens may directly cause piglet facial and sow teat lesions. Swinepox Virus (SWPV) is a pathogen that can cause cutaneous lesions on the piglets' face and also on sows' teats (10, 72). Other pathogens such as Vesivirus, different strains of Picornavirus and mycotoxins may all induce lesions on sows' teats (10).

Based on the survey results, the reported severity of piglet face and sow teat lesions did not differ with farm size (data not shown).

## Discussion

This paper presented the findings of a literature review on the risk factors for piglet facial and sow teat lesions, the two conventional rationales for routine teeth resection in piglets. We integrated these findings with those of the first global online survey on piglet teeth reduction practices in which we collected information on lesion occurrence and the risk factors considered important by pig producers and other relevant stakeholders.

Six main risk factors were identified in the literature, namely teeth resection and housing (farrowing system and flooring), along with litter size, piglet management (including those used to manage large litters, such as cross-fostering and using nurse sows), environmental enrichment and milk production. Most studies found a protective effect of teeth resection to reduce piglet facial lesions but some reported minor and transient effects. The effect of teeth resection on sow teat lesions was less evident. Housing sows in farrowing crates appears to increase the risk of these lesions, so does a large litter size. Some studies looked at the effect of piglet management strategies, but the scarce literature showed inconsistent results. Milk supplementation for piglets can reduce their facial lesions, but our literature review did not find other studies investigating the link between milk production and lesions. Even fewer studies examined the effects of providing environmental enrichment on the lesions.

The survey results showed that half of the respondents practise teeth resection to manage piglet facial lesions and sow teat lesions. When asked about the main reasons for the occurrence of these lesions, leaving piglet teeth intact was closely followed by poor milk production, large litter sizes and a lack of environmental enrichment. In addition, many respondents mentioned other reasons for the occurrence of piglet facial and sow teat lesions, including some of the risk factors mentioned in the literature: management strategies used to manage large litters (cross-fostering: too much or too little) and features of the environment (flooring and farrowing system). These were also indicated as measures that were tried by the respondents to manage these lesions. The responses suggest that there is a gap between the current scientific evidence on the risk factors for piglet facial and sow teat lesions and the practical knowledge of those working in pig farming, who are aware of different risk factors other than those associated with piglets' intact teeth.

### The role of teeth resection

Most studies to date investigated the effect of teeth resection or different methods of teeth resection on piglet facial and sow teat lesions. The survey results also showed that leaving piglets with intact teeth was identified as one of the main causes of these lesions, and such lesions, in turn, were the main justification for practicing teeth resection. As these lesions are the collateral damage during fighting for access to teats, it is understandable that resecting piglets' teeth can have a preventive effect when piglets' natural defences are made less damaging.

However, the survey results also showed a tendency that when teeth resection is practised, other potentially effective measures (e.g., improving sows' milk production, reducing litter size, better litter management practices and changing farrowing housing systems, as discussed below) that could help manage these lesions may be more likely either not used or not considered useful. It is possible that producers tried all other measures to control these lesions but to no avail and decided to resect piglets' teeth as a last resort, which would be in the spirit of the EU legislation. More likely, is that teeth resection became habitual thereby discouraging efforts to try alternative means of addressing the roots of the problem. Many respondents stated that the reason for practising teeth resection was that it had become a “standard procedure”. There is an analogy with tail docking, which is routinely carried out to prevent tail biting, whereas tail docking only masks the underlying issues that cause pigs to tail bite (73): the routine practice of tail docking has created a “system inertia,” i.e., farming practices have become resistant to change (74). Considering that 50% of the respondents to our survey did not practise teeth resection, it would be beneficial to provide scientific support for and further disseminate the practices they employed to prevent piglet facial and sow teat lesions.

Piglets struggled more during teeth resection compared to those undergoing sham-processes, and some studies found that piglets performed more oral behaviours and spent less time playing and fighting after the procedure [as reviewed by (14)]. There is also no definitive evidence showing which method of teeth resection is preferable in terms of animal welfare [reviewed by (12)]. However, the latest scientific opinion on pig welfare suggests that grinding, performed correctly and with the correct equipment, is preferred over clipping should teeth resection be deemed necessary (75). Regardless of the methods used, during teeth resection, the restraint of piglets by securing their mouths in an open position, can interfere with their vocalisations and movement, which can make it more difficult to assess their aversion to this procedure (14). Another study also suggests that there may be a long-term local inflammatory response due to this procedure (76). Considering the potential negative consequences of teeth resection on piglet welfare, the focus on how to prevent piglet facial and sow teat lesions should be shifted to managing other risk factors, as highlighted by our literature review and survey.

### Housing factors

Most survey respondents used farrowing crates and of those who practised teeth reduction, more were using farrowing crates than alternative farrowing systems (although this sample was small). The literature showed that both piglet facial and sow teat lesions were more frequent in crates than in pens, which may explain why a higher proportion of those producers using crates may see this as a problem and may practise teeth reduction. Alternatively, producers using alternative systems may be more likely to practise higher welfare husbandry and not carry out what they see as unnecessary painful procedures. Worker safety may also be a factor, with the caretaker at higher risk of injury from maternally protective sows in open farrowing systems when attempting to carry out piglet processing procedures (77).

Where the farrowing system has fully slatted flooring, more respondents practised teeth reduction, compared to partly slatted or solid flooring. There is no literature to show that flooring *per se* impacts piglet facial lesions, but there is some evidence that solid flooring can have a beneficial impact on sow teat lesions, if it is straw-bedded. Where solid and partly slatted floors are used, the producers may have the option to use straw, which serves the needs of the sow for nest building and provides an outlet for chewing needs of the piglets (65), consequently, with a beneficial impact on lesions and a reduced need for teeth resection.

Since the introduction of farrowing crate in the 1960s (78), it is believed that when the sow was confined in limited space, this would reduce both piglet mortality due to crushing and damage to the teats. However, it is known that in semi-natural conditions, sows remain in the nest with the piglets for the first few days after birth; after that the sows leave the nest for periods of time, in many occasions to socialise with other sows, returning only to suckle the piglets (79, 80). The freedom to get away from the piglets in free farrowing pens and outdoor farrowing systems may be one of the reasons why the piglet facial and sow teat lesions were less pronounced in both the literature and responses from the survey, and therefore the need to resect piglets' teeth. The freedom of movement and avoidance of stress due to confinement may in turn benefit sows' milk production. However, it should be noted that there is a lack of epidemiological data on the prevalence of these lesions in alternative systems as well as in indoor farrowing systems, which calls for further studies.

Although most survey respondents still use conventional farrowing crates, it should be noted that we did include changing the farrowing systems as one of the measures to manage piglet facial and sow teat lesions. As the costs for converting the farrowing system are high, it is not likely that producers would consider changing their farrowing systems simply because of these lesions. However, as a result of the European Citizens' Initiative “End The Cage Age”, the European Commission is committed to put forward by 2023 a legislative proposal to phase out the use of all forms of cages in animal agriculture, including farrowing crates, presumably by 2027 (81). Improved milk production, better nursing and sow-piglet interaction and the reduced risks of piglet facial and sow teat lesions should all be emphasised as benefits of alternative farrowing systems.

### The challenge of large litter sizes

The literature review showed that large litter sizes increase the incidence of fighting among piglets and that they can increase the prevalence of piglet facial and sow teat lesions. Sows' functional teats in relation to litter size can influence the severity of teat disputes and therefore piglet facial lesions, and it is also reported that larger litter size was positively correlated with within-litter birth weight variation (12). Although higher within-litter birth weight variation did not cause more teat disputes directly (48), some studies did find smaller piglets fight longer when their littermates were bigger (12). The survey respondents who did not practise teeth reduction reported more favourably numerically that avoiding large litters helped with coping with the lesion problems. Numerically, the respondents who practised teeth reduction had larger litter sizes, while those who reported reducing litter sizes as a successful preventative measure were the ones that do not practise teeth reduction. However, although the respondents recognised that large litter sizes may increase the occurrence of these lesions, they did not consider that litter size was one of the reasons for resorting to teeth reduction. However, as previously mentioned, this could be due to the fact that once a habitual practice is established, it is more difficult to change (82). There are other recognised animal health and welfare problems in livestock farming for which there does not seem to be sufficient farmers' awareness and/or motivation to change, or alternatively, the limiting factors are prevailing, with one notable example being lameness in dairy cattle (83–85). It is plausible that, also in relation to the disadvantages of large litter sizes in pig farming, knowledge dissemination needs to be improved. Additionally, an approach based on social and cognitive science can be useful to understand the farmers' beliefs and constraints underlying certain decisions (84) and eventually promote changes (85).

The pig industry has genetically selected breeding sows for large litters sizes based primarily on economic considerations, i.e., an increase in pigs produced per sow per year (42, 50). However, Andersen et al. (2) found that piglet mortality due to starvation and crushing increases with litter size; in particular, they suggested that 10–11 piglets are possibly close to the upper limit of what sows can successfully wean. Modern sows may have the capacity to give birth to more piglets (86), but piglet mortality is also positively correlated with litter size (due to the insufficient number of functional mammary glands) as reported by Zimmerman et al. (10). Therefore, an increase in litter size does not directly translate into better efficiency, as pigs weaned per sow per year may not change due to the loss of piglets in large litters, and more labour needs to be put into piglet management which may not be efficient either. For example, cross-fostering lower-weight piglets can improve survivability, but fights will still occur and strategies to maximise weight gain while minimising aggressive bouts should include the careful mixing of small and larger piglets in a litter (48). On the other hand, using foster sows or artificial rearing methods to improve the survival rates of small piglets from large litters may present other animal welfare problems (56). All these considerations should prompt a serious reflection on whether the persistent focus on genetic selection for large litter sizes should be reconsidered (42, 71), for instance, in favour of breeding for resilience (e.g., litter size at weaning rather than at birth), which can also respond to environmental concerns (87). In line with this, the recent Scientific Opinion on pig welfare recommends that to avoid excessive competition for access to teats in large litters, the average number of piglets born alive in a given sow breed or line should not exceed, and preferably be lower than, the average number of functional teats (75).

### The importance of sow milk production

From the literature review, there is a clear knowledge gap for the effect of milk production on piglet facial and sow teat lesions. Given that the survey respondents identified poor milk production as an important risk factor for such lesions, similar to teeth reduction and litter size, there is a need to better understand how variation in milk production impacts these lesions and how to ensure adequate milk production.

Good production of milk involves a number of internal [e.g., hormones (88–90)] and external factors. External factors such as litter size (91), suckling behaviour of the piglets (92), and environmental conditions [e.g., high temperatures (93)] can all influence milk production. Mechanical stimulation by the piglets for at least 2 min is essential for milk let-down as sows have no teat cisterns. Massage of the udder stimulates production of oxytocin which causes the alveolar myoepithelium to contract and eject the milk (94, 95). Illness in the sow, social stress in large litters and poor udder massage (by a weak or very small litter) can all affect the release of oxytocin (94, 96) and therefore milk production/let-down. Teat fidelity is commonly observed, i.e., piglets show preference for specific teat/teat pairs, and anterior or posterior teats are usually preferred by piglets (97, 98). However, the reasons for these preferences are still inconclusive, e.g., heavier piglets did not suckle more on anterior teats (99), and the positive effect of suckling anterior teats on weight gain was only transient (98). As the cause for piglet facial and sow teat lesions can be traced back to teat fighting in order to gain access to milk let-down, it is surprising that no published study investigated the link between the quality of sows' milk production, colostrum quality and the onset of these lesions. Further research should prioritise in filling this gap.

### The role of environmental enrichment

Almost one third of the survey respondents indicated a lack of environmental enrichment or nesting material as a possible reason behind the occurrence of piglet facial or sow teat lesions, and some of them successfully used enrichment to address the issue. Although limited in number, the few studies on this topic validated the idea that effective environmental enrichment can redirect piglet manipulating behaviours away from the sow and littermates and thereby supporting the feasibility of leaving piglets' teeth intact. Providing adequate environmental enrichment is a legal requirement on pig farms in the EU, and it should be stressed that enrichment can contribute to reducing the need for routine painful procedures on piglets, including teeth resection and tail docking (82, 100).

However, it should be noted that the success of enrichment strategies is dependent on the effectiveness of the enrichment provided (100). In Lewis et al. (64), all piglets could access the shredded paper provided as enrichment at the same time (as it could be removed from a box), but only a limited number of piglets could access the rope at any one time. In a study by Pol et al. (101), an algae cylinder provided during the suckling period did not affect lesion scores (although lesion scores only included an ear assessment). The study found that overall, this type of object was of marginal enrichment interest to the pigs, similar to a non-effective metal chain (102).

Telkänranta et al. (103) confirmed the potential benefits in providing enrichment for suckling piglets, although they did not measure piglet facial lesions. The piglets with access to chewable ropes and paper, manipulated (with oro-nasal behaviours) pen mates less frequently than piglets in control pens preweaning (significant in weeks 2 and 3 after birth). Instead, they directed their nose and mouth manipulation at the ropes and paper. These findings are in line with the provisions of the EU Pig Directive, which prescribes the use of adequate environmental enrichment for all pigs as an important means of preventing redirected behaviours and, thus, to avoid teeth reduction and tail docking (17).

### Future research directions and practical recommendations

Although our sample size was small, our survey demonstrated that the respondents working in the pig industry appear to be ahead of the science in trying to solve the issue of piglet facial and sow teat lesions. Just over half of them declared that they are able to manage these lesions without resorting to teeth resection. On the one hand, it is important to facilitate the knowledge exchange between stakeholders in pig production so that more producers can learn about the best practices to prevent these lesions without the need for teeth resection. On the other hand, it is equally important for research to catch up with practice and specifically investigate the direct link between litter size, milk production or environmental enrichment and piglet facial and sow teat lesions and to scientifically validate reportedly successful interventions. Future efforts, including proportionate expansion of information gathered from major pig production sectors and improvement of scientific outreach and dissemination through better producer engagement, offer the opportunity to strengthen background knowledge to support and provide direction in future research endeavours. More science-based knowledge behind the occurrence of these lesions is crucial in putting more focus to identify, analyse and propose ways to address all the risk factors instead of heavily leaning toward teeth resection.

Moreover, it is important to recognise that many of the risk factors identified in this study both by the literature review and survey are interconnected and interdependent. The farrowing system used and litter size may affect milk production and nursing behaviour, and litter size and milk production of the sow may also affect what types of piglet management strategy are needed. There may also be an additive effect of multiple risk factors, e.g., a large litter size in a conventional farrowing system may require more cross-fostering or nurse sow interventions which exacerbates the severity of piglet facial or sow teat lesions. It is therefore imperative to think holistically about these risk factors in relation to each other when providing practical advice to producers.

In conclusion, based on the literature review and the survey results, we recommend future research to fill the knowledge gap of the impact of litter size, farrowing housing system, environmental enrichment and sows' milk quality on piglet facial and sow teat lesions. For producers in the EU and elsewhere, where routine teeth resection is not permissible as a routine practice, selecting suitable farrowing system, keeping litter size manageable, providing meaningful enrichment and checking sows' milk production would be good starting points as alternatives to reduce the risk of these lesions and hence the need for routine teeth resection. It is always worth reiterating that piglet painful husbandry procedures mask underlying problems and do not address their causes while perpetuating suboptimal rearing practices. Research should work together with producers to seek better solutions to the root of these issues.

## Data availability statement

The original contributions presented in the study are included in the article/Supplementary material, further inquiries can be directed to the corresponding author.

## Ethics statement

The survey was conducted in a completely anonymous manner. No personal information was obtained through the survey (e.g., names, emails, IP address, etc. that could have identified individuals), and we asked for explicit consent before respondents could continue to the survey stating, you agree that you're at least 18 years of age and the information you provided will be used for research purposes only.

## Author contributions

The survey was distributed by J-YC, EN, TH, HW, LB, and SI. All authors contributed to the conceptualisation, writing, reading and approval of the final manuscript.

## Funding

This project was an output from the 3Ts Alliance, which is a non-profit voluntary stakeholder group. The publication fee was funded by World Animal Protection.

## Conflict of interest

HW was employed by Cerebrus Associates Ltd. SI was employed by World Animal Protection International. The remaining authors declare that the research was conducted in the absence of any commercial or financial relationships that could be construed as a potential conflict of interest.

## Publisher's note

All claims expressed in this article are solely those of the authors and do not necessarily represent those of their affiliated organizations, or those of the publisher, the editors and the reviewers. Any product that may be evaluated in this article, or claim that may be made by its manufacturer, is not guaranteed or endorsed by the publisher.

## Author disclaimer

The views and opinions expressed in this review do not necessarily reflect the official policy or position of the authors' respective employer or government. Mention of any trade name, proprietary product or specific equipment does not constitute a guarantee or warranty by USDA-ARS and does not imply its approval to the exclusion of other products that may also be suitable. The USDA-ARS is an equal opportunity and affirmative action employer, and all agency services are available without discrimination.
