# Supplementary material for: Investigating risk factors behind piglet facial and sow teat lesions through a literature review and a survey on teeth reduction
Source: Front Vet Sci. 2022 Dec 2;9:909401. doi: 10.3389/fvets.2022.909401 (PMC9755856; doi:10.3389/fvets.2022.909401)
Supplement: Supplementary file 1 [file Table_1.PDF]

Supplementary Material I

Identifying risk factors for piglet facial and sow teat lesions through a literature review and teeth reduction survey

Jen-Yun Chou, Jeremy N. Marchant, Elena Nalon, Thuy Huynh T. T., Heleen A. van de Weerd, Laura A. Boyle, Sarah H. Ison

*** Correspondence:**
Dr Jen-Yun Chou
jenyun.chou@gmail.com

Supplementary material I, Table 1. Full list of search terms used in the literature review

Supplementary material I, Table 1. Full list of search terms used in the literature review

**General search terms:**	
Piglet facial lesion	(sows OR pigs OR swine OR piglets), (face OR facial OR snout or MOUTH) (lesions OR injuries OR damage OR laceration OR wound OR abrasion)
Sow teat lesion	(sows OR pigs OR swine), (teat OR udder), (lesions OR injuries OR damage OR laceration OR wound OR abrasion)

**Risk factors**	**Search terms**
Teeth resection/reduction	(teeth OR tooth)
Housing system	(crate OR pen OR outdoor); floor
Litter size	(litter size); (teat order)
Piglet management	(piglet management); (cross-foster); (nurse sow); (artificial rear); (farrowing management)
Environmental enrichment	(enrichment); (substrate); (manipulable material); (toy)
Milk production	(milk); (mother); (parity); (supplement); (diet); (let down)
