# Supplementary material for: Investigating risk factors behind piglet facial and sow teat lesions through a literature review and a survey on teeth reduction
Source: Front Vet Sci. 2022 Dec 2;9:909401. doi: 10.3389/fvets.2022.909401 (PMC9755856; doi:10.3389/fvets.2022.909401)
Supplement: Supplementary file 2 [file Table_2.PDF]

Supplementary Material II

Identifying risk factors for piglet facial and sow teat lesions through a literature review and teeth reduction survey

Jen-Yun Chou, Jeremy N. Marchant, Elena Nalon, Thuy Huynh T. T., Heleen A. van de Weerd, Laura A. Boyle, Sarah H. Ison

*** Correspondence:**
Dr Jen-Yun Chou
jenyun.chou@gmail.com

Supplementary material II. Full piglet teeth reduction survey (English version)

Piglet teeth reduction survey

We would like to understand the practice of teeth reduction on-farm better. Thank you very much for providing the following information. Your answers will be anonymous. By taking part in this survey, you agree that you're at least 18 years of age and the information you provided will be used for research purposes only. The survey will take less than 10 minutes to complete (20 short questions). Thank you in advance for taking the time to fill out this survey.

This survey is conducted by the "3Ts Alliance," which is a voluntary initiative by various global stakeholders from the industry, academia, NGO and other sectors, who aim to address the issue of tail docking (“T”ail), teeth reduction (“T”eeth) and surgical castration (“T”esticles) to improve animal welfare standards in pig production. Find out more about us at https://www.linkedin.com/company/3ts-alliance

* Required

1. 1. Is teeth reduction (clipping or grinding) routinely practised on your farm? *

Mark only one oval.

- ⬭ Yes *Skip to question 2*
- ⬭ No (to Q5 automatically) *Skip to question 5*

General management practice - teeth reduction

2. 2. Do you use teeth clipping or grinding? (multiple answer possible) *

Check all that apply.

- [ ] Clipping
- [ ] Grinding

3. 3. How often do you practise teeth reduction? *

Mark only one oval.

- ( ) Occasionally when I see problems in some litters, on all piglets in those litters
- ( ) Occasionally when I see problems in some litters but not on small piglets in those litters
- ( ) Frequently on most litters, on all piglets
- ( ) Frequently on most litters, but not on small piglets
- ( ) Always on all litters, on all piglets
- ( ) Always on all litters, but not on small piglets

4. 4. At what age do you carry out teeth reduction of piglets? (multiple answers possible) *

Check all that apply.

- [ ] Within 48 hours after birth
- [ ] From age day 3-7
- [ ] Anytime when I see problems in some litters

Other: [ ] ____________________

General management practice - farrowing

5. 5. What farrowing system do you use? *

Mark only one oval.

- ( ) Conventional farrowing crate
- ( ) Free farrowing pen
- ( ) Outdoor farrowing
- ( ) Other: ____________________

6. 6. What floor type is in your farrowing house? (Please describe the floor area where the sow can lie on, for example, fully-slatted, partly-slatted, concrete, metal, plastic, etc.) *

7. 7. What sow farrowing management topics do you train your farrowing house staff in? (multiple answers possible) *

Check all that apply.

- [ ] Learning about sow farrowing behaviour
- [ ] Checking sow more regularly before farrowing
- [ ] Assisting farrowing
- [ ] Counting number of teats
- [ ] Feed/nutrition adjustment
- [ ] Check water flowrate or consumption
- [ ] Check sow milk production
- [ ] No specific training

Other: [ ] ______________________

8. 8. What types of management strategies are you using/have you used? (multiple answers possible) *

Check all that apply.

- [ ] Cross-fostering
- [ ] Split suckling
- [ ] Use of nurse sows
- [ ] Artificial rearing (e.g. rescue decks)
- [ ] Milk supplementation (via milk cups or similar)
- [ ] None of the above

9. 9. What is your average litter size (born alive)? *

Mark only one oval.

- Less than 10 piglets
- 11-12 piglets
- 13-15 piglets
- 16-18 piglets
- More than 18 piglets

10. 10. Do you provide nesting materials or environmental enrichment in the farrowing crates/pens? *

Mark only one oval.

- Both
- Only nesting materials for sows
- Only environmental enrichment for piglets
- No
- Other: ____________________

Problems and solutions

In this section we are asking questions about the problems and solutions on piglet facial and sow teat lesions.

11. 11. Why do you practise teeth reduction? If you do not practise this, please select reasons based on your past experience or opinion. (multiple answers possible) *

Check all that apply.

- [ ] Standard procedure
- [ ] Ease of overall management in the farrowing house
- [ ] Problems with piglet facial lesions
- [ ] Problems with sow teat injuries
- [ ] Too large litter sizes
- [ ] Insufficient milk production

Other: [ ] ______________________

12. 12. Could you describe the severity of the problems on your farm? *

Mark only one oval per row.

	Never	Manageable without needing to change management practices	Needed to adjust management practices to keep it manageable	Not manageable
Piglet facial lesions	◯	◯	◯	◯
Sow teat lesions	◯	◯	◯	◯

13. 13. What do you think are the main reasons for the occurrence of these problems? (multiple answers possible)

Check all that apply.

	Piglet facial lesions	Sow teat lesions
Piglet’s teeth are clipped or ground	☐	☐
Piglet’s teeth are NOT clipped or ground	☐	☐
Large litter size	☐	☐
Using conventional farrowing crates	☐	☐
Using free farrowing pens	☐	☐
Outdoor farrowing	☐	☐
Flooring in the farrowing accommodation	☐	☐
Poor milk production of sows	☐	☐
Not enough cross-fostering	☐	☐
Too much cross-fostering	☐	☐
Lack of environmental enrichment/nesting material	☐	☐

14. 14. What measures have you tried other than teeth reduction to solve your problems, and which ones worked/did not work? (multiple answers possible)

Check all that apply.

	I have tried this	Worked	Did not work
Only used teeth reduction	☐	☐	☐
Avoid large litter size (keep litter size at around/below 12-13 piglets)	☐	☐	☐
Select for sows with good mother traits	☐	☐	☐
Improve sow nutrition at farrowing	☐	☐	☐
Check on sows more frequently	☐	☐	☐
Increase sow water intake	☐	☐	☐
Provide early supplementary piglet nutrition	☐	☐	☐
Frequent cross-fostering	☐	☐	☐
Split suckling	☐	☐	☐
Use nurse sows	☐	☐	☐
Artificial rearing	☐	☐	☐
Provide nesting material/enrichment in the farrowing crate/pen	☐	☐	☐

15. 15. If you have used any other measures that are not listed above, please specify here and give your opinion of whether or not it worked.

Basic information

16. 16. In which country are you based in? *

Mark only one oval.

- Afghanistan
- Albania
- Algeria
- Andorra
- Angola
- Antigua and Barbuda
- Argentina
- Armenia
- Australia
- Austria
- Azerbaijan
- Bahamas
- Bahrain
- Bangladesh
- Barbados
- Belarus
- Belgium
- Belize
- Benin
- Bhutan
- Bolivia
- Bosnia and Herzegovina
- Botswana
- Brazil
- Brunei
- Bulgaria
- Burkina Faso
- Burundi
- Cabo Verde
- Cambodia

Cambodia

Cameroon

Canada

Central African Republic (CAR)

Chad

Chile

China

Colombia

Comoros

Congo, Democratic Republic of the

Congo, Republic of the

Costa Rica

Cote d'Ivoire

Croatia

Cuba

Cyprus

Czechia

Denmark

Djibouti

Dominica

Dominican Republic

Ecuador

Egypt

El Salvador

Equatorial Guinea

Eritrea

Estonia

Eswatini (formerly Swaziland)

Ethiopia

Fiji

Finland

France

Gabon

Gambia

- [illegible]
- Georgia
- Germany
- Ghana
- Greece
- Grenada
- Guatemala
- Guinea
- Guinea-Bissau
- Guyana
- Haiti
- Honduras
- Hungary
- Iceland
- India
- Indonesia
- Iran
- Iraq
- Ireland
- Israel
- Italy
- Jamaica
- Japan
- Jordan
- Kazakhstan
- Kenya
- Kiribati
- Kosovo
- Kuwait
- Kyrgyzstan
- Laos
- Latvia
- Lebanon
- Lesotho

- [illegible]
- Liberia
- Libya
- Liechtenstein
- Lithuania
- Luxembourg
- Madagascar
- Malawi
- Malaysia
- Maldives
- Mali
- Malta
- Marshall Islands
- Mauritania
- Mauritius
- Mexico
- Micronesia
- Moldova
- Monaco
- Mongolia
- Montenegro
- Morocco
- Mozambique
- Myanmar (formerly Burma)
- Namibia
- Nauru
- Nepal
- Netherlands
- New Zealand
- Nicaragua
- Niger
- Nigeria
- North Korea
- North Macedonia (formerly Macedonia)

North Macedonia (formerly Macedonia)

Norway

Oman

Pakistan

Palau

Palestine

Panama

Papua New Guinea

Paraguay

Peru

Philippines

Poland

Portugal

Qatar

Romania

Russia

Rwanda

Saint Kitts and Nevis

Saint Lucia

Saint Vincent and the Grenadines

Samoa

San Marino

Sao Tome and Principe

Saudi Arabia

Senegal

Serbia

Seychelles

Sierra Leone

Singapore

Slovakia

Slovenia

Solomon Islands

Somalia

South Africa

- South Africa
- South Korea
- South Sudan
- Spain
- Sri Lanka
- Sudan
- Suriname
- Sweden
- Switzerland
- Syria
- Taiwan
- Tajikistan
- Tanzania
- Thailand
- Timor-Leste
- Togo
- Tonga
- Trinidad and Tobago
- Tunisia
- Turkey
- Turkmenistan
- Tuvalu
- Uganda
- Ukraine
- United Arab Emirates (UAE)
- United Kingdom (UK)
- United States of America (USA)
- Uruguay
- Uzbekistan
- Vanuatu
- Vatican City (Holy See)
- Venezuela
- Vietnam
- Yemen

[illegible]

Zambia

Zimbabwe

Other

17. 17. How many sows are kept in your herd? *

Mark only one oval.

Under 10

10 to 99

100 to 999

1000 and over

18. 18. What is your role on the farm? *

Mark only one oval.

Stockperson (responsibility covering farrowing house)

Stockperson (responsibility NOT including farrowing house)

Farm manager

Farm owner

Other: ________________

19. 19. What is your gender? *

Mark only one oval.

Female

Male

Prefer not to say

Other: ________________

20. 20. What is your age group? *

Mark only one oval.

- Below 30
- 30-39
- 40-49
- 50-59
- Above 60
- Prefer not to say

21. Please provide any additional information that you deem relevant. If you would like to be contacted about the outcomes of the study, please email 3Tsalliance@gmail.com . We also have a teeth reduction fact sheet that can be downloaded on our LinkedIn page https://tinyurl.com/y9aor65f. Thank you for your participation!

This content is neither created nor endorsed by Google.

Google Forms
