# Supplementary material for: Investigating risk factors behind piglet facial and sow teat lesions through a literature review and a survey on teeth reduction
Source: Front Vet Sci. 2022 Dec 2;9:909401. doi: 10.3389/fvets.2022.909401 (PMC9755856; doi:10.3389/fvets.2022.909401)
Supplement: Supplementary file 3 [file Table_3.PDF]

Supplementary Material III

Identifying risk factors for piglet facial and sow teat lesions through a literature review and teeth reduction survey

Jen-Yun Chou, Jeremy N. Marchant, Elena Nalon, Thuy Huynh T. T., Heleen A. van de Weerd, Laura A. Boyle, Sarah H. Ison

*** Correspondence:**
Dr Jen-Yun Chou
jenyun.chou@gmail.com

Supplementary material III: Additional survey results

Supplementary material III Table 1. Demographics of the respondents (*N* = 75).

Respondent demographics	%	n
Country		
France	14.7	11
Ireland	13.3	10
Thailand	13.3	10
Netherlands	12.0	9
United Kingdom	8.0	6
Portugal	6.7	5
Spain	6.7	5
Brazil	5.3	4
Australia	5.3	4
China	4.0	3
Italy	2.7	2
Bahamas	1.3	1
Canada	1.3	1
Chile	1.3	1
Mexico	1.3	1
Philippines	1.3	1
Taiwan	1.3	1
Gender		
Male	66.7	50
Female	30.7	23
Prefer not to indicate	2.7	2
Age		
Below 30	13.3	10
Between 30 and 39	33.3	25
Between 40 and 49	26.7	20
Between 50 and 59	21.3	16
Over 60	4.0	3
Preferred not to say	1.3	1
Role on the farm		
Farm owner	38.7	29
Veterinarian	21.3	16
Farm manager	16.0	12
Stockperson	12.0	9
Directors	2.7	2
Working on quality	2.7	2
Technical roles	2.7	2
Science/research roles	2.7	2
Supply chain coordinator	1.3	1
Farm size (no. of sows)		
Under 10	2.7	2
10 to 99	8.0	6
100 to 999	48.0	36
1000 or over	41.3	31

Supplementary material III Table 2. The reported reasons for the occurrence of piglet facial (face) and sow teat (teat) lesions by the reported severity of those lesions.

		Severity				
**Reason for lesion**	**Lesion type**	Never	Manageable without needing to change management practices	Needed to adjust management practices to keep it manageable	Not manageable	Total
Teeth not reduced	Face	4	20	10	2	36 (48.0%)
	Teat	7	18	10	2	37 (49.3%)
Poor milk production	Face	6	21	9	0	36 (48.0%)
	Teat	5	17	12	0	34 (45.3%)
Large litters	Face	4	25	6	0	35 (46.7%)
	Teat	6	16	7	0	29 (38.7%)
Lack of enrichment	Face	5	12	4	0	21 (28.0%)
	Teat	2	4	7	0	13 (17.3%)
Too much cross fostering	Face	3	11	5	0	19 (25.3%)
	Teat	1	6	3	0	10 (13.3%)
Low cross fostering	Face	1	9	3	0	13 (17.3%)
	Teat	0	5	2	0	7 (9.3%)
Flooring	Face	2	3	1	0	6 (8.0%)
	Teat	2	5	4	0	11 (14.7%)
Teeth are reduced	Face	2	6	2	0	10 (13.3%)
	Teat	0	6	1	0	7 (9.3%)
Using crates	Face	1	1	3	0	5 (6.7%)
	Teat	0	4	4	0	8 (10.7%)
Using free farrowing	Face	2	0	1	0	3 (4.0%)
	Teat	1	2	0	0	3 (4.0%)
Using outdoor farrowing	Face	1	0	1	0	2 (2.7%)
	Teat	2	1	1	0	4 (5.3%)

Supplementary material III Table 3. The reported severity of sow teat (teat) and piglet facial (face) lesions according to whether or not teeth reduction is currently practiced.

**Could you describe the severity of the problems on your farm?** (select one for teat and face injuries)	**Teeth reduction**		
	**No**	**Yes**	**All**
Teat: Never	11 (14.7)	6 (8.0)	17 (22.7)
Teat: Manageable without changing management practices	20 (26.7)	18 (24.0)	38 (50.7)
Teat: Needed to adjust management practices	6 (8.0)	12 (16.0)	18 (24.0)
Teat: Not manageable	1 (1.3)	1 (1.3)	2 (2.6)
Face: Never	15 (20.0)	3 (4.0)	18 (24.0)
Face: Manageable without changing management practices	19 (25.3)	20 (26.7)	39 (52.0)
Face: Needed to adjust management practices	3 (4.0)	13 (17.4)	16 (21.3)
Face: Not manageable	1 (1.3)	1 (1.3)	2 (2.7)

Supplementary material III Table 4. The total number of answers selected by respondents for the questions that allowed multiple answers is shown (left) by whether or not teeth reduction is practised with the effect size (W) and *P*-value for the differences.

Question (and answer options)	Statistic	Teeth reduction No (n = 38)	Teeth reduction Yes (n = 37)
What sow farrowing management topics do you train your farrowing house staff in? (learning about sow farrowing behaviour, checking sow more regularly before farrowing, assisting farrowing, counting number of teats, feed/nutrition adjustment, check water flowrate or consumption, check sow milk production)	Median	5	5
	Mean	4.29	4.49
	Min - Max	0 - 7	0 - 7
	W, *P*-value	646.5, 0.546	
What types of management strategies are you using/have you used**?** (cross-fostering; split suckling; use of nurse sows; artificial rearing (e.g. rescue decks); milk supplementation (via milk cups or similar))	Median	2.5	3
	Mean	2.24	2.63
	Min - Max	0 - 5	0 - 5
	W, *P*-value	612.5, 0.330	
What do you think are the main reasons for the occurrence of these problems [facial lesions]? (piglet's teeth are reduced; piglet's teeth are NOT reduced; large litter size; using farrowing crates; using free farrowing pens; outdoor farrowing; flooring in the farrowing accommodation; poor milk production; not enough cross-fostering; too much cross-fostering; lack of enrichment/nesting material)	Median	2	2
	Mean	2.76	2.19
	Min - Max	0 - 9	0 - 9
	W, *P*-value	769.5, 0.477	
What do you think are the main reasons for the occurrence of these problems [teat lesions]? (piglet's teeth are reduced; piglet's teeth are NOT reduced; large litter size; using farrowing crates; using free farrowing pens; outdoor farrowing; flooring in the farrowing accommodation; poor milk production; not enough cross-fostering; too much cross-fostering; lack of enrichment/nesting material)	Median	2	2
	Mean	2.29	2.05
	Min - Max	0 - 9	0 - 7
	W, *P*-value	718.5, 0.871	
What measures have you tried to solve your problems? (only teeth reduction; avoid large litters (at around/below 12-13 piglets); select sows with good mother traits; improve sow nutrition; check sows more frequently; increase sow water intake; early supplementary piglet nutrition; frequent cross-fostering; split suckling; nurse sows; artificial rearing; provide nesting material/enrichment)	Median	4	6
	Mean	5.08	5.22
	Min - Max	0 - 11	0 - 11
	W, *P*-value	684.5, 0.848	
What measures have you tried to solve your problems [and selected 'worked']? (only teeth reduction; avoid large litters (at around/below 12-13 piglets); select sows with good mother traits; improve sow nutrition; check sows more frequently; increase sow water intake; early supplementary piglet nutrition; frequent cross-fostering; split suckling; nurse sows; artificial rearing; provide nesting material/enrichment)	Median	1.5	0
	Mean	2.47	1.43
	Min - Max	0 - 11	0 - 9
	W, *P*-value	855, 0.042	

Supplementary material III Table 5. Reported farrowing system and floor type according to whether or not teeth reduction is currently practiced.

**Question** and options	Is teeth reduction practiced on the farm?		
	No	Yes	All
**What farrowing system do you use?**			
Conventional farrowing crate	28	35	63
Free farrowing pen	6	1	7
Outdoor farrowing	4	1	5
**What floor type is in your farrowing house?**			
Fully or partly slatted	1	1	2
Fully slatted	11	18	29
Partly slatted	18	8	26
Solid	4	1	5
Concrete	9	2	11
Metal	3	9	12
Plastic	2	5	7
Pasture	2	1	3
Metal and concrete	4	4	8
Metal and plastic	5	7	12

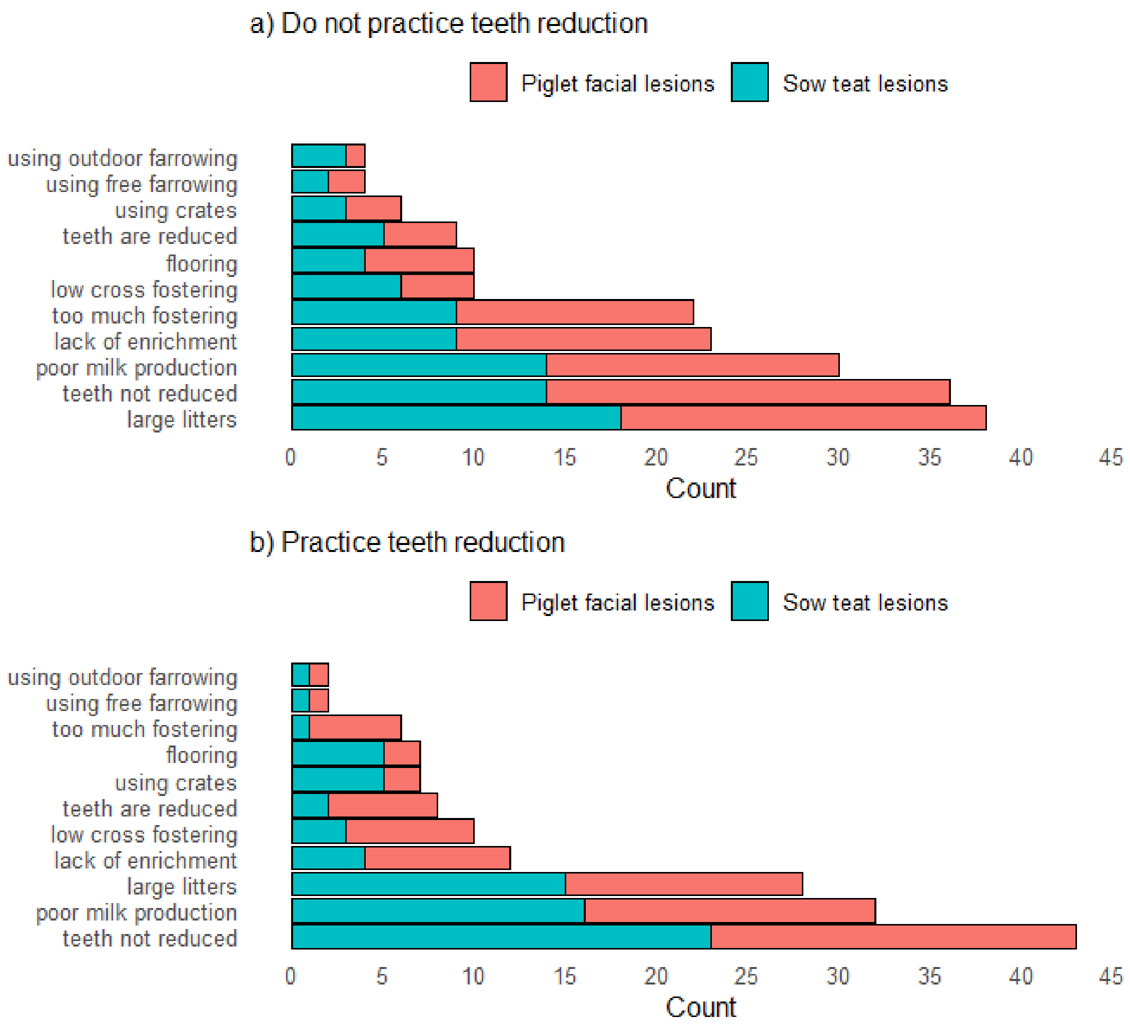

Supplementary material III Figure 1. The reported reasons behind the occurrence of piglet facial and sow teat lesions between those who practise teeth reduction or not.
